# Preparation, Characterization, and Mechanism of Antifreeze Peptides from Defatted Antarctic Krill (*Euphausia superba*) on *Lactobacillus rhamnosus*

**DOI:** 10.3390/molecules27092771

**Published:** 2022-04-26

**Authors:** Yu Liu, Xuena Yu, Yanling Zhu, Wei Yang, Yan Zeng, Yi Hu, Wei Jiang

**Affiliations:** 1 Key Laboratory of Key Technical Factors in Zhejiang Seafood Health Hazards, Institute of Innovation & Application, Zhejiang Ocean University, Zhoushan 316022, China; liuyu1987@zjou.edu.cn (Y.L.); yxnycn@163.com (X.Y.); 13037665057@163.com (Y.Z.); 18434162948@163.com (Y.Z.); 2 Rizhao Agricultural Products Quality and Safety Inspection and Testing Center, Rizhao 276800, China; brightgirl8619@163.com; 3 Zhejiang Henghe Food Co., Ltd., Zhoushan 316022, China; huyi@hailisheng.com

**Keywords:** antifreeze peptide, Antarctic krill, by-products, cryoprotectant, *Lactobacillus rhamnosus*, freezing

## Abstract

Defatted Antarctic krill powder is the main by-product in the manufacturing of krill oil. Exploring a high value-added approach for utilizing this protein-rich material has received much attention in research and industry. Given this, the preparation and primary characterization of antifreeze peptides from defatted Antarctic krill (AKAPs) were carried out in this study. The cryoprotective effect of AKAPs on *Lactobacillus rhamnosus* ATCC7469 was also investigated. The results showed that Protamex was the optimum protease for AKAP preparation from defatted Antarctic krill. AKAPs were found to be rich in short peptides, with the MW ranging from 600 to 2000 Da (69.2%). An amino acid composition analysis showed that AKAPs were rich in glutamic acid (18.71%), aspartic acid (12.19%), leucine (7.87%), and lysine (7.61%). After freezing, the relative survival rate of *Lactobacillus rhamnosus* in the 1.0 mg/mL AKAP-treated group (96.83%) was significantly higher than in the saline group (24.12%) (*p* < 0.05). AKAPs also retarded the loss of acidifying activity of *L. rhamnosus* after freezing. AKAPs showed even better cryoprotective activity than three commercial cryoprotectants (sucrose, skim milk, and glycerol). In addition, AKAPs significantly alleviated the decrease in *β*-galactosidase and lactic dehydrogenase activities of *L. rhamnosus* (*p* < 0.05). Furthermore, AKAPs effectively protected the integrity of *L. rhamnosus* cell membranes from freezing damage and alleviated the leakage of intracellular substances. These findings demonstrate that AKAPs can be a potential cryoprotectant for preserving *L. rhamnosus*, providing a new way to use defatted Antarctic krill.

## 1. Introduction

Antarctic krill (*Euphausia superba*) is an excellent marine biological resource, due to its enormous biomass and great nutritional value [1]. The total biomass of Antarctic krill is estimated to be approximately 3.8 × 10^9^ million tons [2,3]. As reported, Antarctic krill contains 77.9–83.1% moisture, 11.9–15.4% crude protein, 0.5–3.6% lipids, and approximately 2% chitin [4,5]. Recently, krill oil has become a popular dietary supplement on the market, due to its ability to reduce blood triglyceride levels, prevent the pathological injuries of diabetic cardiomyopathy, and alleviate methamphetamine-induced memory impairment [6,7,8]. Krill oil is usually extracted from dried Antarctic krill powder by organic solvent. As a result, a considerable amount of defatted Antarctic krill has been generated from krill oil manufacturing. Defatted Antarctic krill powder is a cheap by-product with over 65–75% protein (based on its dry weight) [1]. There is an urgent need to develop high-valued uses for defatted Antarctic krill to conserve resources and prevent environmental pollution.

Lactic acid bacteria (LAB) transform carbohydrates into lactic acid under appropriate conditions. They are widely used as probiotics and starter cultures in fermented foods [9,10]. *Lactobacillus rhamnosus* is one of the most commonly used probiotics. It can produce only *L*-lactic acid during fermentation, without producing undesired acids that may affect the taste and safety of foods [11]. The commercialization of LAB demands preservation methods that maximize its survival, activity, and shelf life. Freezing is a commonly used technique to maintain LAB’s cellular and desired technological properties. However, the cellular membrane is always sensitive to low temperatures. Freezing may cause cellular structure damage, cellular viability deterioration, and acidifying activity attenuation [12]. These undesired side effects could be alleviated to some extent by the usage of cryoprotectants [13]. Nowadays, skim milk, sucrose, phosphates, glycerol, and dimethyl sulfoxide (DMSO) are widely used as cryoprotectants [14]. However, some disadvantages of these cryoprotectants have attracted much attention in the food industry. For instance, traditional antifreeze agents, such as skim milk, sucrose, and phosphates, may result in an undesired taste, high fat content, and high energy, while organic agents, such as DMSO, may cause irreversible cytotoxic damage to probiotics cells [15,16]. Hence, innovative antifreeze agents that can overcome the above shortcomings should be developed.

Antifreeze peptides are a category of protein hydrolysates that can protect frozen products from cold damage [17]. Antifreeze peptides show many typical properties, including thermal hysteresis ability (THA) [17], and the ability to non-colligatively reduce the freezing point of solutions [18], inhibit the growth of crystals by binding to the surface of ice crystals [19], inhibit recrystallization [20], and reduce cell damage [21]. Antifreeze peptides have a broad application prospect in the frozen food system, such as in dough, surimi, and LAB [15,22,23,24]. It has been reported that collagen hydrolysates from shark skin, pig skin, and tilapia scales have been used successfully to improve the survival of cold-treated LAB [19,25,26].

This study aimed to prepare antifreeze peptides from defatted Antarctic krill through enzymatic hydrolysis. Defatted Antarctic krill was hydrolyzed by five commercial proteases. The molecular weight (MW) distribution and amino acid composition of the obtained antifreeze peptides from defatted Antarctic krill (AKAPs) were characterized. Furthermore, the cryoprotective activity and mechanism of AKAPs on *L. rhamnosus* ATCC7469 were also investigated.

## 2. Results and Discussion

### 2.1. Preparation of Antifreeze Peptides from Defatted Antarctic Krill by Enzymatic Hydrolysis

**Figure 1:**
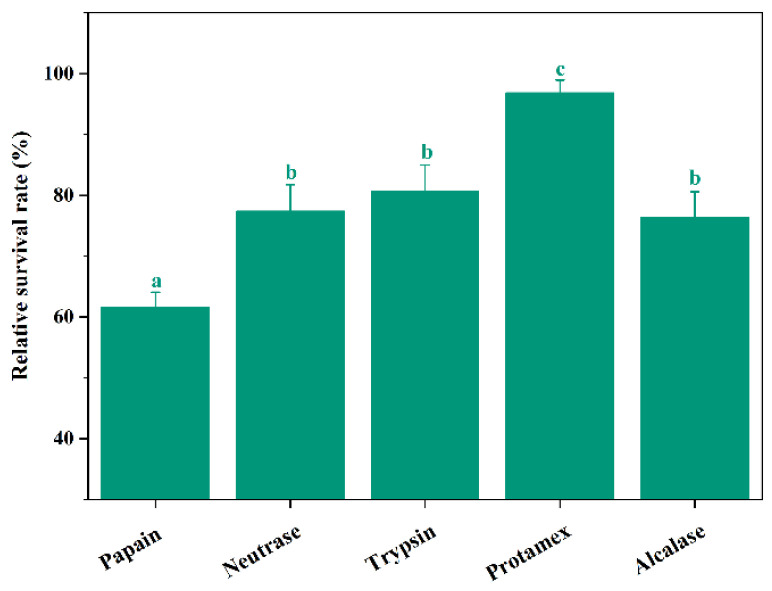
Cryoprotective activities of enzymatic hydrolysates from defatted Antarctic krill at a 1.0 mg/mL concentration treated with different proteases. Bars with different lowercase letters (a, b, and c) indicate significant differences (*p* < 0.05).

The hydrolysis site was determined by the specificity of protease, which affected the composition and biological activity of the enzymatic hydrolysate [27]. Thus, defatted Antarctic krill was hydrolyzed by five commercial proteases, separately (Neutrase, Alcalase, Papain, Trypsin, and Protamex), and the cryoprotective activities (measured as described in Section 3.3) of five corresponding enzymatic hydrolysates are shown in Figure 1. At a concentration of 1.0 mg/mL, the relative survival rate of Protamex hydrolysate was 94.83 ± 2.12%, which is significantly higher than those of Dispase, Alcalase, Papain, and Trypsin hydrolysates (*p* < 0.05). The lowest relative survival rate was observed in Papain hydrolysate (61.66 ± 2.43%). Therefore, the antifreeze peptides from defatted Antarctic krill (named AKAPs) were hydrolyzed with Protamex for further research. Protamex was also used for the enzymolysis of surimi processing by-products, green crab, and skin gelatin [28,29,30].

### 2.2. Molecular Weight Distribution of AKAPs

**Figure 2:**
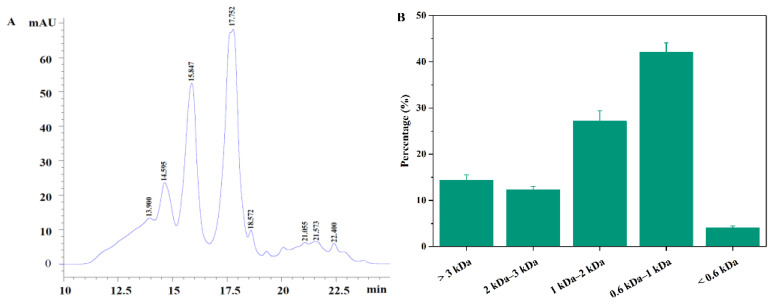
(**A**) HPLC elution of molecular weight and (**B**) molecular weight distribution of antifreeze peptides from defatted Antarctic krill (AKAPs).

The MW distribution of AKAPs, as measured by HPLC, is shown in Figure 2A. AKAPs were rich in short peptides, with the MW ranging from 600 to 2000 Da, which comprised 69.2% AKAPs (Figure 2B). The results suggest that AKAPs mainly consisted of peptides with 5–20 amino acids, which was similar to the MW distribution of antifreeze peptides from tilapia scales [14]. A previous study revealed that short peptides played a critical role in cryoprotective activity by easily adsorbing onto the ice crystal’s surface and inhibiting the growth of ice crystallization [31].

### 2.3. Amino Acid Compositions of AKAPs

The amino acid composition of AKAPs is shown in Table 1. AKAPs were found to be rich in glutamic acid (18.71%) and aspartic acid (12.19%), followed by leucine (7.87%), lysine (7.61%), alanine (6.72%), and arginine (6.04%). These amino acids were relative to the ice affinity and cryoprotective activity of the antifreeze proteins [14,18,19,32,33]. Furthermore, the alkyl side chains of alanine and leucine residues in AKAPs provided a non-polar environment. They maintained the hydrogen bonds formed by the hydrophobic interaction with water molecules, thus inhibiting ice crystal growth [17]. A previous study showed that antifreeze peptides obtained from the tilapia scale contained 24.41% glycine, 12.80% proline, 12.43% glutamic acid, and 10.46% alanine [14].

### 2.4. Cryoprotective Activity of AKAPs on L. rhamnosus

**Figure 3:**
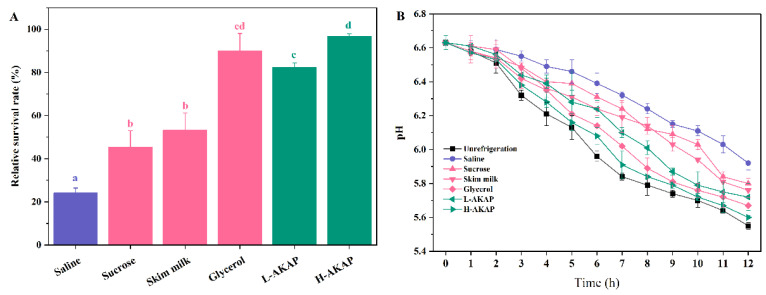
Effects of the negative control (saline), positive controls (1 mg/mL sucrose, 1 mg/mL skim milk, and 20% glycerol), 0.5 mg/mL antifreeze peptides from defatted Antarctic krill (AKAP) (L-AKAP), and 1 mg/mL AKAP (H-AKAP) on the (**A**) growth and (**B**) pH values of *L. rhamnosus* after freezing. The unrefrigeration group in (**B**) did not experience the freezing process. Bars with different lowercase letters (a, b, c, and d) indicate significant differences (*p* < 0.05).

Freezing and freeze-drying were widely used to preserve probiotics, but a loss of viability and activity was consistently observed during storage [34]. After freezing, the cryoprotective activity of AKAPs on *L. rhamnosus* is shown in Figure 3A. The relative survival rates of the L-AKAP (0.5 mg/mL) and H-AKAP (1.0 mg/mL) groups were 82.33% and 96.83%, respectively, which were significantly higher than those of the negative group (saline, 24.12%) and the two positive groups (1 mg/mL sucrose and 1 mg/mL skim milk) (*p* < 0.05). Sucrose and skim milk have been commonly used as commercial cryoprotectants in the food industry to protect probiotics. In this study, AKAPs exhibited better cryoprotective activity than sucrose and skim milk. The relative survival rate of the positive control group (20% glycerol) reached 90.06%, which is lower than that of the H-AKAP group (96.83%).

The acidifying activity was important for the functioning of lactic acid bacteria [9]. As shown in Figure 3B, the pH values of the different groups decreased significantly when cultured in a skim milk medium (*p* < 0.05). The speed at which the pH values declined represented the acidifying activity of *L. rhamnosus*. The acidifying activity of *L. rhamnosus* in the unrefrigeration group was the highest, and was inhibited by freezing. The trend of acidifying activity with different treatments of *L. rhamnosus* after freezing was similar to that of the growth. As the incubation time increased, the H-AKAP group showed the lowest pH values among six freezing-treated groups. The results indicate that AKAPs effectively retarded the loss of acidifying activity of *L. rhamnosus* during freezing. The protection of the acidifying activity of *Streptococcus thermophilus* by antifreeze peptides obtained from tilapia scales and snow flea has also been reported previously [14,15].

### 2.5. β-Galactosidase (β-GAL) and Lactic Dehydrogenase (LDH) Activities of L. rhamnosus

**Figure 4:**
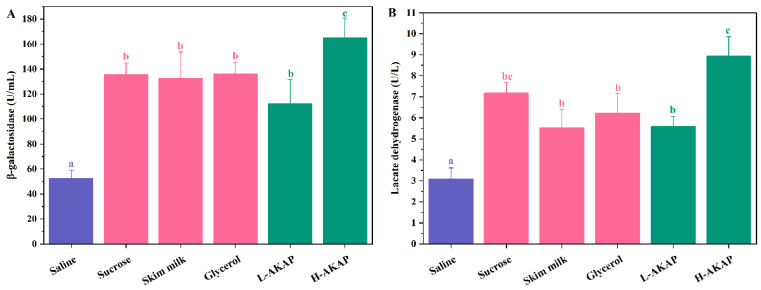
Effects of the negative control (saline), positive controls (1 mg/mL sucrose, 1 mg/mL skim milk, and 20% glycerol), 0.5 mg/mL antifreeze peptides from defatted Antarctic krill (AKAPs) (L-AKAP), and 1 mg/mL AKAP (H-AKAP) on the (**A**) *β*-galactosidase (*β*-GAL) activity and (**B**) lactic dehydrogenase (LDH) activity of *L. rhamnosus* after freezing. Bars with different lowercase letters (a, b, and c) indicate significant differences (*p* < 0.05).

*β*-GAL catalyzes the hydrolysis of lactose to glucose and galactose, which is an important enzyme for the probiotic effects of lactic acid strains [35]. The *β*-GAL activity of *L. rhamnosus* before freezing was 184.00 U/mL (data not shown in Figure 4). The effects of saline and various cryoprotectants on the *β*-GAL activities of *L. rhamnosus* are shown in Figure 4A. The *β*-GAL activity of the negative control (saline) was 52.43 U/mL, which was significantly lower than those of the other groups (*p* < 0.05). The highest *β*-GAL activity was observed in the H-AKAP group (164.97 U/mL) (89.66% of the *L. rhamnosus* before freezing). No significant differences were observed among the three positive groups and the L-AKAP group (*p* > 0.05), which showed *β*-GAL activities ranging from 112.33 U/mL to 136.12 U/mL. The results suggested that the *β*-GAL activity of *L. rhamnosus* decreased after freezing. Nevertheless, AKAPs were able to protect the β-GAL activity of *L. rhamnosus* during hypothermia.

LDH catalyzes the reduction of pyruvate to lactic acid in the metabolism of lactic acid strains, which is sensitive during the freezing process [36,37]. The LDH activity of *L. rhamnosus* prior to freezing was 11.49 U/L (data not shown in Figure 4). The LDH activities of *L. rhamnosus* analyzed after freezing are shown in Figure 4B, which suggests that the LDH activity was impaired after freezing. Three commercial cryoprotectants and AKAPs significantly alleviated the reduction of LDH activity (*p* < 0.05). The highest LDH activity of 8.95 U/L was observed in the H-AKAP group (77.89% of *L. rhamnosus* before freezing), while that of the saline group was only 3.09 U/L. The results indicate that AKAPs are able to maintain the LDH activity of *L. rhamnosus* during freezing.

The protective effect of AKAPs on *β*-GAL and LDH activities might be attributed to the stabilization of cell membranes and the prevention of intracellular ice formation, which is similar to the antifreeze mechanism of sucrose [38]. A similar effect on *β*-GAL and LDH activities was observed in antifreeze peptides prepared from pig skin collagen and tilapia scales [14,19].

### 2.6. Cell Membrane Permeability

**Figure 5:**
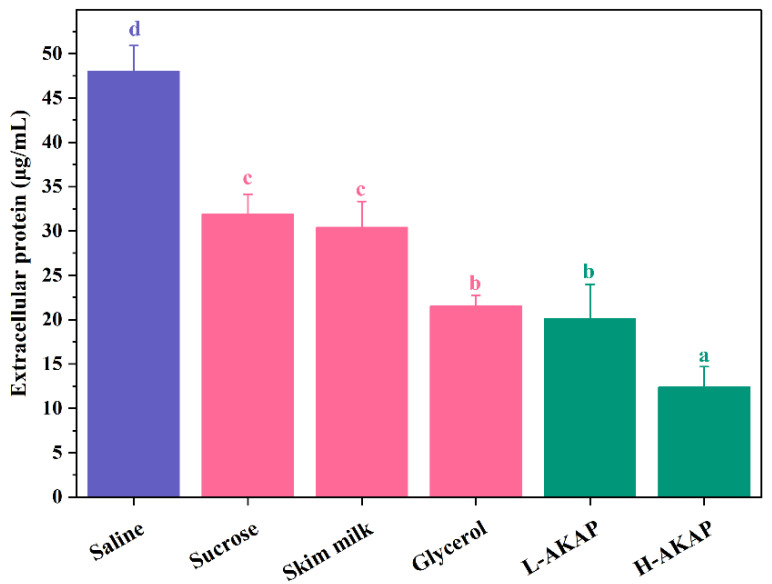
Effects of the negative control (saline), positive controls (1 mg/mL sucrose, 1 mg/mL skim milk, and 20% glycerol), 0.5 mg/mL antifreeze peptides from defatted Antarctic krill (AKAPs) (L-AKAP), and 1 mg/mL AKAP (H-AKAP) on concentrations of extracellular protein of *L. rhamnosus* after freezing. Bars with different lowercase letters (a, b, c, and d) indicate significant differences (*p* < 0.05).

The cell membrane was sensitive to temperature change and was also the primary damage target during freezing [18]. The destruction of cell membranes causes the leakage of intracellular substances, such as proteins and nucleic acids [19]. Thus, the concentration of extracellular protein after freezing was measured to reflect the integrity of *L. rhamnosus* cells. As shown in Figure 5, the highest extracellular protein concentration was found in the saline group (48.01 μg/mL). The extracellular protein concentrations of the L-AKAP and H-AKAP groups were 20.12 μg/mL and 12.40 μg/mL, respectively, which were both lower than those of the three positive groups. The results revealed that freezing caused severe damage to the *L. rhamnosus* cell membrane, without the addition of cryoprotectants. Nevertheless, AKAPs might effectively protect the integrity of *L. rhamnosus* cell membranes from freezing damage. Previous studies have also shown that antifreeze peptides obtained from tilapia scales and antifreeze glycopeptide analogues obtained by nonenzymatic glycation could reduce the leakage of intracellular substances of lactic acid bacteria [14,18].

### 2.7. Morphological Characterization

**Figure 6:**
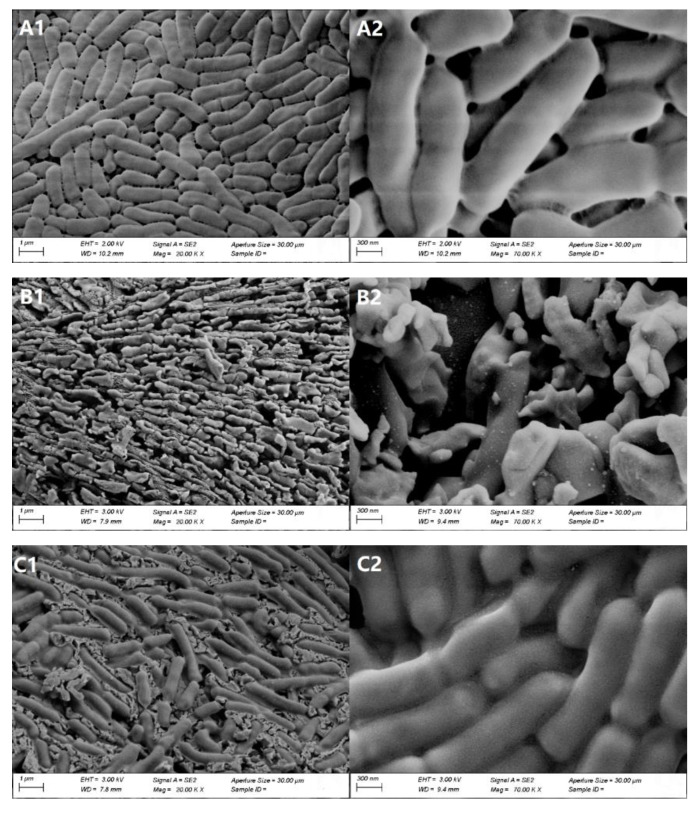
Representative scanning electron micrographs of (**A**) unrefrigerated *L. rhamnosus*, (**B**) saline-treated *L. rhamnosus* after freezing, and (**C**) *L. rhamnosus* treated with 1 mg/mL antifreeze peptides from defatted Antarctic krill (AKAPs) (H-AKAP) after freezing. (**1**) represents 20,000-fold magnifications and (**2**) represents 70,000-fold magnifications.

Scanning electron microscopy was performed to display the protection effect of AKAPs on freezing-induced damage to *L. rhamnosus* cells, and the results are shown in Figure 6. Figure 6A1,A2 shows the cell morphological images of *L. rhamnosus* without freezing. These cells had a regular shape with integrated cell walls and clear boundaries, and no debris was found in the surrounding environment. After freezing treatment with saline, the cells had severe ruptures, and many pieces of debris were observed in the entire field of vision (Figure 6B1,B2). This suggests that freezing resulted in the cell membrane rupture of *L. rhamnosus*, which caused the leakage of intracellular substances. The intracellular substances were deposited on the debris of cells, forming unclear boundaries. With the treatment with 1.0 mg/mL AKAP, the cells were covered by thin layers of the protective glassy matrix formed by AKAP (Figure 6C1,C2). Debris and extracellular compounds were also observed, but clearly decreased. These observations confirm the results in Figure 5. Furthermore, the results indicate that AKAPs effectively alleviate the damage to *L. rhamnosus* cells’ structural integrity and the leakage of intracellular substances.

## 3. Materials and Methods

### 3.1. Materials

Defatted Antarctic krill powder was provided by Zhejiang Hailisheng Biotechnology Co., Ltd. (Zhoushan, China), with a proximate composition as follows: moisture (8.29%), protein (78.33%), fat (1.17%), sugar (0.84%), and ash (4.62%). Neutrase (5 × 10^4^ U/g), Alcalase (2 × 10^5^ U/g), Papain (1 × 10^5^ U/g), Trypsin (2.5 × 10^5^ U/g), Protamex (1 × 10^5^ U/g), the BCA protein assay kit, the *β*-GAL assay kit, and the LDH assay kit were obtained from Beijing Solarbio Science & Technology Co., Ltd. (Beijing, China). *L. rhamnosus* ATCC7469 was provided by Shanghai Luwei Microbial Sci. &Tech. Co., Ltd. (Shanghai, China). MRS broth and MRS agar were purchased from Qingdao Hope Bio-Technology Co., Ltd. (Tsingtao, China). The other reagents and chemicals were of analytical grade.

### 3.2. Preparation of Antifreeze Peptides from Defatted Antarctic Krill Powder

The defatted Antarctic krill powder was mixed with 0.05 mol/L phosphate buffer (1:10, *w/v*). The mixtures were hydrolyzed for 6 h, separately, by Neutrase, Alcalase, Papain, Trypsin, and Protamex under optimum enzymatic conditions with a dosage of 2000 U/g (enzyme activity-to-substrate). Afterward, five hydrolysates were kept in a boiling water bath for 15 min to inactivate the enzymes, and were centrifuged at 7155× *g* and 4 °C for 10 min. The supernatants were collected and freeze-dried for further tests.

### 3.3. Determination of Cryoprotective Activity

The cryoprotective activity was assayed according to a pre-defined method [18], with minor modifications to the culture medium and some experimental parameters. Briefly, *L. rhamnosus* was activated in an MRS broth medium at 37 °C for 24 h. The strain was sub-cultured in MRS medium with the incubation of 2% (*v/v*) seed medium at 37 °C. Cells were obtained during the logarithmic phase by centrifugation (7155× *g* and 4 °C for 10 min). The obtained sediment was washed twice with sterile saline, and the cells’ density was adjusted to OD_600_ (optical density at 600 nm) of 1.0 using sterile saline. The cell suspension (0.5 mL) was transferred to a 2 mL centrifuge tube, which contained 0.5 mL of a test sample. These centrifuge tubes were frozen at −20 °C for 24 h and thawed at 37 °C for 15 min after storage. Then, 0.1 mL of each sample was transferred into 1.9 mL of MRS broth. The mixture was cultured at 37 °C for 20 h. The relative survival rate of *L. rhamnosus* was calculated as the ratio (expressed as a percentage) of the absorbance at 600 nm of the microbial cultures incubated with the *L. rhamnosus* cell mixture before and after freezing.

Sterilized saline was used as a negative control. Positive controls included sucrose (1 mg/mL), skim milk (1 mg/mL), and glycerol (20%, *v*/*v*).

### 3.4. Determination of Molecular Weight (MW) Distribution

The MW distribution was analyzed using an HPLC system (Agilent 1260, Santa Clara, CA, USA) equipped with a TSKgel 2000SWXL column and VWD detector [39]. The mobile phase (acetonitrile:water:TFA = 40:60:0.05, *v*/*v*/*v*) was monitored at 220 nm with a flow rate of 0.5 mL/min. The standards used for establishing the relative molecular weight correction curve included cytochrome C (MW 12,355 Da), aprotinin (MW 6511 Da), bacitracin (MW 1422 Da), Gly-Gly-Tyr-Arg (MW 451 Da), and Gly-Gly-Gly (MW 189 Da).

### 3.5. Amino Acid Composition

The freeze-dried samples were treated with 6 mol/L HCl at 110 °C for 24 h under a nitrogen atmosphere. The digested mixtures were transferred to centrifuge tubes and dried under reduced pressure at 50 °C. The dried samples were dissolved twice with the addition of distilled water. Finally, the dried samples were dissolved in a sodium citrate buffer (pH 2.2) and filtrated through 0.22 µm filters. The amino acid composition was analyzed by an amino acid analyzer (HITACHI L-8900, Tokyo, Japan) [40].

### 3.6. Determination of Acidifying Activity

The acidifying activity of *L. rhamnosus* was assayed according to the previously reported method [14], with minor modifications to the culture method (static culture instead of shake culture). Briefly, the microbial culture tubes containing 4 mL skim milk medium (85 g/L powder) were sterilized at 110 °C for 20 min. Then, 0.05 mL of 10^−3^-fold gradient dilution thawed microbial mixture was added and incubated at 37 °C for 12 h. The pH values of the microbial cultures were measured every one hour.

### 3.7. Determination of β-GAL and LDH Activities

The cell-free extracting solutions were prepared according to the published method [19], with minor modifications to some experimental parameters. Five milliliters of the *L. rhamnosus* cell culture was centrifugated at 7155× *g* and 4 °C for 10 min. The cells were collected after being washed twice with sterile saline. Subsequently, the cells were suspended in an equal volume of testing sample solutions. The cell suspensions were frozen at −20 °C for 24 h and thawed at 37 °C for 15 min. The thawed cells were centrifuged at 7155× *g* and 4 °C for 10 min and washed twice with sterile saline. The obtained cells were suspended in sterile saline, and the cells’ density was adjusted to OD_600_ of 1.0. The cell suspensions were disrupted by a BILON-100 ultrasonic cell disruptor (Shanghai Bilon Instrument Co., Ltd., Shanghai, China) in an ice bath for 120 cycles of 2 s at 200 W. The cell-free extracting solutions were collected by centrifugation at 4 °C and 11,180× *g* for 10 min. The cell-free extracting solutions were subjected to *β*-GAL and LDH activity measurements using a *β*-GAL assay kit and an LDH assay kit.

### 3.8. Determination of Extracellular Protein

The sample treatment was conducted according to a reported method [14], with minor modifications to some experimental parameters. Briefly, five milliliters of *L. rhamnosus* cell culture was centrifugated at 7155× *g* and 4 °C for 10 min. The sediments were obtained, which were washed twice with sterile saline. Subsequently, the cells were suspended in centrifuge tubes with testing sample solutions to the OD_600_ of 1.0. These centrifuge tubes were frozen at −20 °C for 24 h. Two freeze–thaw cycles were performed at 2 h intervals from the start time (thawed at 37 °C for 15 min). These final thawed cell suspensions were centrifugated at 7155× *g* and 4 °C for 10 min. The extracellular protein contents of supernatant fractions were measured using the BCA protein assay kit.

### 3.9. Scanning Electron Microscopy (SEM)

Samples including unrefrigerated cells and treated cells after freezing at −20 °C for 24 h were freeze-dried using a vacuum freeze drier (FD-1C-80, Beijing Biocool Experimental Instrument Co., Ltd., Beijing, China). The freeze-dried cells were covered with a small amount of gold. Microscopy was conducted using a Zeiss Sigma 300 scanning electron microscope (Carl Zeiss, Oberkochen, Germany) [40].

### 3.10. Statistical Analysis

Statistical analyses were performed using the IBM SPSS Statistics 26 software (SPSS Inc., Chicago, IL, USA). Data were analyzed by ANOVA with the Tukey test. All the measurements were carried out in triplicate, and the data were expressed as average ± standard deviation.

## 4. Conclusions

AKAPs were prepared from defatted Antarctic krill using Protamex. During the freezing of *L. rhamnosus*, AKAPs exhibited better cryoprotective activity than commercial cryoprotectants (sucrose, skim milk, and glycerol) and retarded the loss of acidifying activity. In addition, AKAPs protected the β-GAL and LDH activities of *L. rhamnosus* during hypothermia. Furthermore, AKAPs effectively maintained the cell structure and alleviated the leakage of intracellular protein. The study provided a value-added approach for the application of defatted Antarctic krill. The study also elucidated the protective mechanism of AKAPs on *L. rhamnosus*; the former could, thus, potentially be used as a cryoprotectant in the low-temperature storage of probiotics.

## Figures and Tables

**Table 1:** Amino acid compositions of antifreeze peptides from defatted Antarctic krill (AKAPs).

Amino Acid	Molar Percentage (%)	Amino Acid	Molar Percentage (%)
Glutamic acid	18.71	Isoleucine	4.4
Aspartic acid	12.19	Phenylalanine	4.29
Leucine	7.87	Threonine	4.25
Lysine	7.61	Proline	4.02
Alanine	6.72	Tyrosine	3.76
Arginine	6.04	Methionine	3.03
Glycine	5.14	Histidine	1.56
Valine	5.14	Cysteine	0.57
Serine	4.43		

## Data Availability

Not applicable.

## Sample Availability

Samples of the inclusion peptides are available from the authors.
